# Is Gut Microbiota a Key Player in Epilepsy Onset? A Longitudinal Study in Drug-Naive Children

**DOI:** 10.3389/fcimb.2021.749509

**Published:** 2021-12-03

**Authors:** Camilla Ceccarani, Ilaria Viganò, Emerenziana Ottaviano, Maria Gaia Redaelli, Marco Severgnini, Aglaia Vignoli, Elisa Borghi

**Affiliations:** ^1^Institute of Biomedical Technologies, National Research Council, Segrate, Italy; ^2^Epilepsy Centre, Azienda Socio Sanitaria Territoriale (ASST) Santi Paolo Carlo, University of Milan, Milan, Italy; ^3^Department of Health Sciences, University of Milan, Milan, Italy; ^4^Child Neurology and Psychiatry Unit, Azienda Socio Sanitaria Territoriale Grande Ospedale Metropolitano (ASST GOM) Niguarda, Milan, Italy

**Keywords:** epilepsy, gut microbiota, seizures, inflammation, *Akkermansia*, Proteobacteria, anti-seizure medication

## Abstract

Microbiota alterations have been recently investigated in individuals with epilepsy and in other neurological diseases as environmental factors that play a role, by acting through the gut-brain axis, in the pathological process. Most studies focus on the contribution of bacterial communities in refractory epilepsy and suggest a beneficial role of ketogenic diet in modulating the gut microbiota and seizure occurrence. However, they do not evaluate whether epilepsy itself alters the gut microbiota in these patients or if the gut microbial communities could contribute as a seizure trigger. In this pilot study, we performed 16S rRNA sequencing and investigated the gut microbial communities of eight children at their seizure onset and after anti-seizure was started (one year follow-up) and we compared microbial data with seven healthy children, age- and sex-matched. In drug-naive subjects, we observed a microbial signature that shared several features with those reported in refractory epilepsy, such as an increased abundance in *Akkermansia* spp. and Proteobacteria and a decreased relative abundance in *Faecalibacterium* spp.We suggest that a bacterial-mediated proinflammatory milieu could contribute to seizure occurrence in children with new onset of epilepsy, as already reported for individuals with drug-resistant epilepsy, and that it could vary during treatment in those who are drug-responsive.

## Introduction

The microbiota-gut-brain axis has recently gained growing interest as a new frontier for explaining the complex features of different neurological diseases (Borghi and Vignoli, 2019; Cryan et al., 2020). In the field of epilepsy, recent studies have hypothesized that the gut microbiota could contribute to maintain an inflammatory state that could potentially drive drug-resistant seizures (Dahlin and Prast-Nielsen, 2019; De Caro et al., 2019).

Despite the large proliferation of studies in animal models examining the link between an altered microbial composition and epilepsy (Medel-Matus et al., 2018; Olson et al., 2018; De Caro et al., 2019; Citraro et al., 2021), studies in humans are still scarce. (Lum et al., 2020). Patients with epilepsy can become seizure-free with the appropriate use of anti-seizure medications (ASMs). However, ASMs have different sites of action, with different molecular mechanisms that could potentially lead to drug resistance. Although several different therapeutic approaches are currently available for patients with drug-resistant epilepsy (DRE), seizure control cannot be achieved in many subjects (Dalic and Cook, 2016). Therefore, it is important to search for new complementary therapeutic strategies that can influence the clinical picture and improve the patient’s quality of life.

In the last few years, the discovery of the potential contribution of the microbiota in our gastrointestinal system to central nervous system diseases has opened many research and future opportunities (De Caro et al., 2019; Ma et al., 2019). The central and the nervous systems of the gastrointestinal tract are tightly connected by hormones, neuromodulators, and neurotransmitters related to efferent/afferent nerves including the vagus nerve (Grenham et al., 2011). Since most of the microorganisms inhabiting our body are gastrointestinal residents, these microbes are perfectly situated to react to and influence neuronal, humoral, metabolic, or immune signaling underlying the gut-brain relationship.

Despite the possible impact of the gut-brain axis mechanisms on neurological diseases, limited information is available in literature about the composition of the intestinal microbiota in patients with epilepsy and on how this could be linked to seizures or to ASMs (Simrén et al., 2013; Dahlin and Prast-Nielsen, 2019). In particular, there is no information about the composition of gut microbiota in drug-naive patients at epilepsy onset. The present study aims at describing the longitudinal evolution of gut microbiota in a group of children newly diagnosed with epilepsy and followed during subsequent ASMs treatment, thus providing new baseline information for future investigations.

## Materials and Methods

### Patient Selection

We enrolled children (aged 3-16 years) admitted to ASST Santi Paolo e Carlo Hospital in Milan (Italy) after their first seizures. Inclusion criteria were non-lesional focal or generalized epilepsy and neurotypical development. Exclusion criteria were chronic or acute intestinal diseases, special diets, and treatments with antibiotics or probiotics within the three months before enrollment.

We collected fecal samples at the time of enrollment and at 4 (T4) and 12 (T12) months after the introduction of ASMs. As a control group, we included 7 mentally and physically healthy children, age- and sex-matched, who were not on any medications. A 3-day dietary survey was completed by the caregivers at the time of enrollment. The diary included three consecutive days, one of which was during the weekend. Children were asked to maintain their usual eating pattern, and caregivers were instructed to record everything including drinks, the cooking method, and the amount of food. Dietary food records were processed using commercially available software (MètaDieta, METEDA srl, Italy). To monitor the children’s gastrointestinal function, caregivers were asked to describe and categorize feces according to the Bristol Stool Form Scale (BSFS, Lewis and Heaton, 1997).

We created a dedicated database that included information about gender, family history for epilepsy and/or febrile seizures, epileptic features, and neurologic examination results. Regarding the epileptic phenotype, we evaluated the type of seizures at onset and at last follow-up according to the ILAE 2017 classification, drug therapy, and electroencephalographic (EEG) pattern at onset and at last follow-up.

Psychomotor and cognitive development was evaluated by formal neuropsychological testing (if available) or best clinical assessment (based on developmental milestones and academic achievement). Brain MRI and metabolic findings were also included in the database.

The study was approved by the Local Ethics Committee (protocol number 2016/ST/199, 28 July 2016). Written informed consent was obtained from the parents and/or legal guardians of the enrolled patients/healthy subjects.

### Gut Microbial DNA Extraction and Sequencing

Fecal DNA extraction was performed using the Spin stool DNA kit (Stratec Molecular, Berlin, Germany), according to the manufacturer’s instructions. The V3–V4 hypervariable regions of the bacterial 16S rRNA gene were amplified with a two-step barcoding approach according to the Illumina 16S Metagenomic Sequencing Library Preparation (Illumina, San Diego, CA, USA). Briefly, DNA samples were amplified with dual-index primers using a Nextera XT DNA Library Preparation Kit (Illumina), while library concentration and quantification were determined using a KAPA Library Quantification Kit (Kapa Biosystems, Woburn, MA, USA) and Agilent 2100 Bioanalyzer System (Agilent, Santa Clara, CA, USA), respectively. The libraries were pooled and sequenced with a MiSeq platform (Illumina) for 2 × 250 base paired-end reads and a total of 2.5 Gbases raw reads were obtained.

### Microbiota Profiling and Bioinformatic Analysis

The obtained 16S rRNA gene paired sequences were merged using Pandaseq (release 2.5; Masella et al., 2012). Reads were filtered by trimming stretches of 3 or more low-quality bases (quality < 3) and discarding the trimmed sequences whenever they were shorter than 75% of the original one. Bioinformatic analyses were conducted using the QIIME pipeline (release 1.9.0; Caporaso et al., 2010), clustering filtered reads into Operational Taxonomic Unit (OTUs) at 97% identity level and discarding singletons as well as possible chimeras. Taxonomic assignment was performed *via* the RDP classifier (Wang et al., 2007) against the SILVA database (release 132; Pruesse et al., 2007) from phylum to genus level. Alpha-diversity was computed through the QIIME pipeline using the Chao1, the number of OTUs (“observed species” index), Shannon diversity, and Faith’s Phylogenetic Diversity whole tree (PD whole tree) metrics. To compare the microbial community structure of the subjects for the beta-diversity analysis, weighted and unweighted UniFrac distances were used. A functional prediction analysis of the bacterial metabolic pathways has been performed through the PICRUSt software (v 1.0.1) (Langille et al., 2013) and KEGG pathways database (Kanehisa, 2004).

### Statistical Analysis

Statistical evaluation among alpha-diversity indices was performed by a non-parametric Monte Carlo-based test in the QIIME pipeline. The Permanova test (adonis function) in the R package vegan (version 2.0-10; Oksanen et al., 2013) was used to determine differences between the dataset cohorts through the beta-diversity analysis. Taxonomic and functional differences were computed and established through the non-parametric Mann-Whitney U-test; the Bonferroni correction has been applied to statistical results. Correlation analysis between nutritional and taxonomic data was conducted using Pearson’s rank correlation coefficients. All analyses have been conducted using R (version 3.6.3 *via* RStudio, version 1.2.1335).

P-values below 0.05 were considered significant among all comparisons and analyses.

## Results

### Cohort Description

We enrolled 8 drug-naive children (6 females, 75%; 2 males, 25%) with epilepsy onset between 3 and 13 years of age. Mean age at epilepsy onset was 8.9 ± 4.3 years. As control group, we collected fecal samples from 7 healthy subjects (healthy control, HC), who were age- and sex-matched (4 females, 66%; 3 males, 33%; mean age 8.0 ± 4.2 years; 5 delivered vaginally and 2 *via* cesarean-section).

Epilepsy diagnosis was Focal epilepsy in 4 subjects, Childhood Absence Epilepsy in 3 subjects, and Adolescence Absence Epilepsy in one. Brain MRIs were unremarkable in all patients and EEGs were consistent with the epilepsy diagnosis. In children with Focal epilepsy, the etiology was undetermined.

Twenty-two fecal samples were collected over the two years of study, with a three time-point scheme: the samples collected at seizure onset and before starting ASMs were named “drug-naive” (DN); the samples obtained 4 months after initiating treatment were labeled as “drug-therapy 4” (DT4); 6 out of 8 patients provided an additional sample after 12 months (labeled “drug-therapy 12”, DT12). Drug therapy was initiated after at least two seizures and titrated according to the clinical practice. All the patients were drug-responders and did not experience epileptic seizures during the experimental year of sampling and observation. Metabolic testing was negative for all patients. All clinical data are summarized in **Table S1**.

According to the Bristol Stool Form Scale (BSFC), none of the children was neither severely constipated nor suffering from diarrhea. Bristol scale was established at DN and remained unchanged at DT4 and DT12 for 7/8 patients; one patient reported a shift from type 2 to type 3 after therapy assumption.

None of the caregivers reported children gastrointestinal discomfort during the 12-month follow-up.

The dietary survey showed no differences in intake of macronutrients. Nutritional parameters are detailed in the relative supplementary table (**Supplementary Material**, **Table S2**).

### Biodiversity Assessment Between Subjects

**Figure 1:**
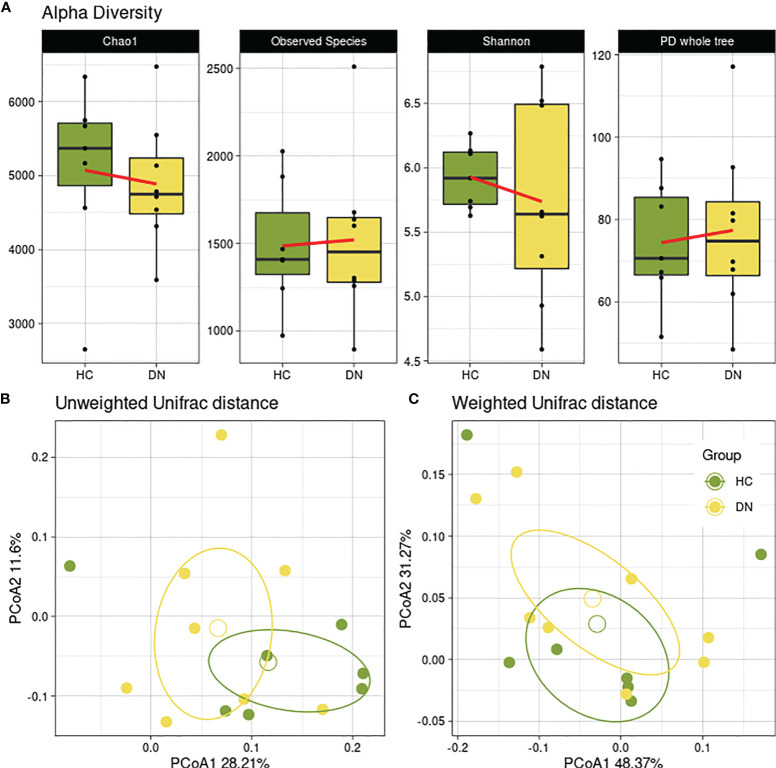
Gut microbial diversity of healthy controls and drug-naive patients. Healthy subjects (HC) were used as controls for epileptic children with drug-naive gut microbiota (DN) sampled after the first seizure. **(A)** Boxplots showing the alpha-diversity measures within 4 metrics (Chao1, Observed Species, Shannon, PD whole tree). No statistical differences were found. Beta-diversity was observed through the Principal Coordinate Analysis of the unweighted **(B)** and weighted **(C)** Unifrac matrix of dissimilarity. The first and second principal coordinates are reported for both measures. Comparisons not significant (p=0.824; p=0.248, respectively).

Initially, possible differences between healthy controls and children experiencing seizures were investigated (HC vs DN). Data showed a reduced trend of bacterial abundances and biodiversity across the Chao1 and Shannon alpha-diversity metrics (**Figure 1A**). Beta-diversity reported a separation as well, in both Unifrac weighted (**Figure 1B**) and unweighted matrices (**Figure 1C**), even though it was not significant.

**Figure 2:**
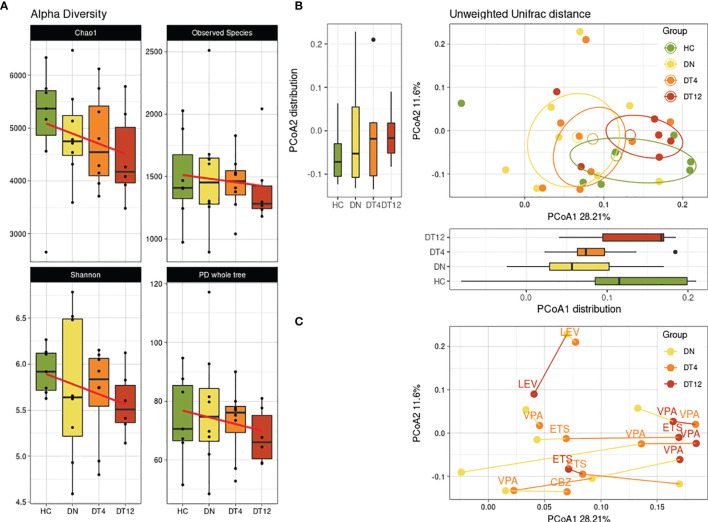
Bacterial diversity over time. Bacterial biodiversity of children with epilepsy at the enrolment (DN), after 4 months (DT4) and after 12 months (DT12) of drug therapy was compared to healthy subjects (HC). **(A)** Alpha-diversity metrics: Chao1, Observed Species, Shannon, PD whole tree. The linear regression computed shows the conditional mean trend along the therapy assumption in time. **(B)** Unweighted beta-diversity of the 4 groups are reported, with mean centroids and confidence ellipses. Principal coordinates 1 and 2 are represented with corresponding distribution boxplots. For both **(A, B)** plots, no significant values were observed. **(C)** Unweighted beta-diversity of subjects in time. The lines connect the samples from each patient, while the colors indicate the time-point of sample collection. Labels show which ASM was taken by the patient at DT4 and DT12 sampling: VPA, Valproic Acid; LEV, Levetiracetam; CBZ, Carbamazepine.

We then considered possible changes in the microbiota composition of children with epilepsy before and after the ASM introduction. A decreasing trend was observed in the alpha-diversity analysis, for all metrics, showing how the children’s gut microbial biodiversity was gradually and constantly reduced over the months of pharmacological treatment (**Figure 2A**).

The trend along samplings was observed also in the beta-diversity analysis among groups (**Figure 2B**). After 12 months of ASM treatment (DT12), we observed that the gut microbial communities of patients showed similarities to those of the HC subjects.

Considering each patient signature across the months of observation, we observed an overall shift along the first principal coordinate of the beta-diversity metric for most samples: 5/8 patients (62.5%) have their DN time-point at lower PCoA1 values, while 4/6 patients (66.7%) have the DT12 samples at the higher values (**Figure 2C**). Interestingly, all the patients who were on valproic acid (VPA) at DT12 were found to be closer to one another at the highest portion of the PCoA1 axis.

### Taxonomic Differences in Children With Epilepsy Through Time

**Figure 3:**
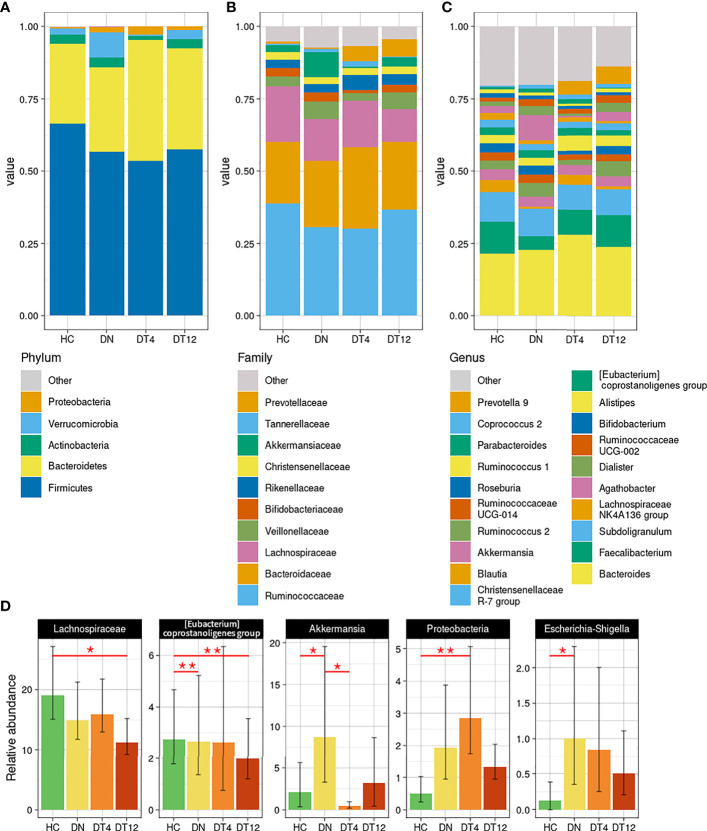
Taxonomy analysis. Bacterial composition has been characterized at the phylum **(A)**, family **(B)**, and genus **(C)** phylogenetic levels of the healthy controls and patients. The main bacterial groups, with a relative abundance higher than 1% among all the groups, are represented for each plot; those with less than the set threshold were clustered into the “Other” group. **(D)** Statistically different bacteria, as detailed in **Table S3**. Mean and standard deviation are represented; p-values < 0.05 were considered significant: * <0.05; **<0.01.

At phylum level (**Figure 3A**), *Proteobacteria* was found significantly increased at DT4 compared to HC (0.5% versus 2.9%, p=0.0137). On the contrary, the relative abundance of *Verrucomicrobia* increased substantially in the DN group with statistical significance when compared to HC (p=0.028) and to DT4 (p=0.012).

Among the main abundant families (**Figure 3B**), *Lachnospiraceae* and *Akkermansiaceae* were found significantly altered between the cohorts: *Lachnospiraceae* decreased steadily in the epileptic patients, from DN to DT12 (respectively, 14.9% and 11.2% compared to HC, 19.1%; p=0.025 HC *vs* DT12); *Akkermansiaceae*, instead, spiked at DN (8.7%, p=0.028 *vs* HC, 2.1%; p=0.012 *vs* DT4, 0.4%).

At genus level (**Figure 3C**), the peak of *Akkermansia* in patients at seizure onset was confirmed from the higher phylogenetic level (8.7% vs 2.1% in HC, p=0.0462). After 4 months of drug intake, this relative abundance dropped rapidly (0.5% at DT4) and, after 12 months, returned to an amount comparable to the abundance found in HC (3.2% at DT12). On the other hand, *Faecalibacterium* relative abundance was decreased in DN (4.7% vs 11% in HC), gradually returning to a normal level after the drug therapy (8.6% at DT4, 11.1% at DT12). ‘*Prevotella 9’* showed the opposite trend: it was nearly absent before the therapy (1.0% in DN patients) and increased after 4 months of drug therapy (4.7% at DT4, 5.9% at DT12) to reach the level seen in HC (4%).

Changes in taxonomic composition across time and during ASM therapy in epileptic patients are detailed in **Table S3** and significant bacterial groups are summarized in **Figure 3D**.

The Bristol score was 3 for all the patients (6/8) but two, who reported a mild constipation (Bristol score = 2) at DN, a condition that recovered at DT12 for one child.

Grouping together samples with BSFC 2 and with BSFC 3, we highlighted two bacterial genus that significantly differ. In particular, *‘Prevotella 9’*, given the high abundance in one patient with BSFC 2 (14.77% compared to 0.01 in BSFC 3) was found significantly more abundant (p=0.004). *‘Coprococcus 2’*, as well, was found more abundant in patients with Bristol score 2 (3.30 vs 0.82 in BSFC 3, p=0.021). Taxonomic abundances depending on the stool type are shown in **Figure S1**.

To investigate the ratio between Gram-positive and Gram-negative bacteria, the first 100 genera identified in the cohorts, corresponding to 99.99% of the cumulative abundance of the total bacterial genera, were analyzed. Children with epilepsy showed a higher abundance of Gram-negative bacteria, even after ASM therapy, compared with HC (**Supplementary Material**, **Figure S2**).

### Functional Prediction

We used PICRUSt to predict possible pathways enriched or depleted in bacterial communities of children with epilepsy; 6,909 KO genes were analyzed. At KEGG functional level 3, the analysis predicted a significant enrichment in genes encoding enzymes for the “Bacterial secretion system” pathways in the microbiota of DN patients compared to HC (0.65% vs 0.56%, respectively; adjusted p-value=0.034), whereas genes involved in “Energy metabolism” pathways were increased at the beginning of ASM therapy (0.92% in DT4 compared to 0.85% in DN, adjusted p-value=0.029). Although not significantly, the predictive metabolic pathways showed a slight increase in the abundance of genes encoding enzymes involved in LPS-related functions in epileptic patients at all time points compared to that of the control group. In particular, the genes encoding for “Lipopolysaccharide biosynthesis proteins” were 0.32% in HC and 0.38%, 0.35%, 0.36% in DN, DT4, and DT12, respectively, and genes involved in “Lipopolysaccharide biosynthesis pathways” were 0.22% in HC and 0.27%, 0.24%, and 0.26% in children with epilepsy (DN, DT4, and DT12, respectively). Predicted gene abundances are reported in the **Supplementary Figure S3**.

### Nutritional Correlation With Bacterial Relative Abundances

**Figure 4:**
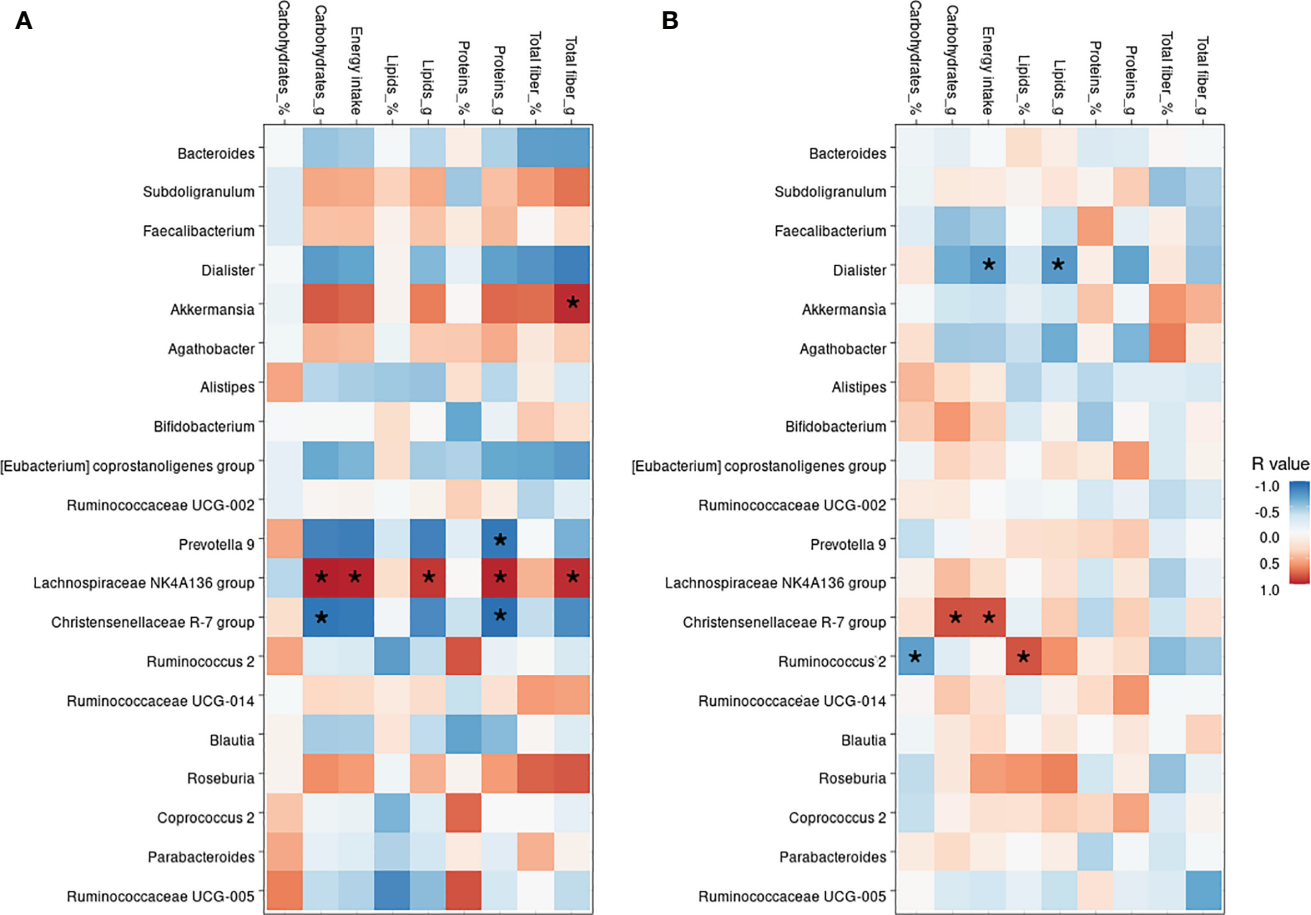
Correlation between nutritional values and bacterial genera. Heatmap showing the Pearson’s rank correlation between HC subjects **(A)** and drug-naive patients, DN **(B)**. In both, * indicate significant p-values, as <0.05.

Although dietary macronutrient intakes were found not statistically different among HC and DN subjects, the correlation analysis between the diet and the most abundant bacterial taxa revealed some divergent relationships in the two groups (**Figure 4**).

The *Christensenellaceae R-7 group* was negatively correlated in the HC group with carbohydrates (p=0.030), energy intake (p=0.040), and proteins (p=0.016), whereas was positively correlated in the DN subjects with carbohydrates (p=0.006) and energy intake (p=0.008). *Akkermansia* was only found positively correlated to the total fiber (p=0.012) intake in HC, as no dietary correlations were observed in DN subjects. On the other hand, *Dialister* was only found negatively correlated with total lipids (p=0.035) and energy intake (p=0.039) only in the DN group. The *Lachnospiraceae NK4A136 group*, which was found profoundly depleted in the DN subjects, was positively correlated with lipids (p=0.021), fibers (p=0.013), carbohydrates (p=0.001), proteins (p=0.006), and energy intake (p=0.004) in HC, whereas no correlations were shown for children with epilepsy.

## Discussion

To our knowledge, this is the first study investigating the possible contribution of the gut microbial communities to the onset of seizures in children. We chose to include in the study individuals with neuro-typical development in order to limit potential confounding factors, such as neurological/behavioral symptoms or genetic disorders. Indeed, most of the available studies on the role of gut microbiota in epilepsy focus on patients with DRE and on the effects of ketogenic diet effects in seizure control (Xie et al., 2017; Zhang et al., 2018; Lindefeldt et al., 2019; Gong et al., 2021; Lee et al., 2021). Moreover, due to the high variability in the enrolled cohorts (i.e. age, body mass index, modes of delivery, type of diet, and control groups), observations regarding microbiota changes in epilepsy are often controversial (Holmes et al., 2020).

In our study, we show a trend towards a reduction in the alpha-diversity, i.e. in the richness and in the evenness of microbial taxa, in children at the onset of seizures. This is in agreement with the observations by Gong and coworkers (Gong et al., 2021), who reported a reduction in the alpha diversity indexes of patients with epilepsy compared with the household control group. Similar data were reported in children with DRE (Lindefeldt et al., 2019). A reduction in biodiversity has been reported as one of the first signs of gut microbiota alterations (Mosca et al., 2016), and has been observed in neurodevelopmental diseases characterized by seizures as a comorbidity (Borghi and Vignoli, 2019; Iannone et al., 2019).

Although all the subjects enrolled in our cohort happened to suffer from drug-sensitive epilepsy (DSE), we found that, at seizure onset and before starting the drug therapy, they shared some gut microbiota signatures with subjects with refractory epilepsy. For instance, a significant increase in the relative abundance of Verrucomicrobia was observed. Previous studies showed an increase in *Akkermansia* in both children and adults with epilepsy (Huang et al., 2019; Gong et al., 2020; Lee et al., 2021). *Akkermansia* is a genus involved in mucin degradation at the gut mucous layer, commonly constituting 1 to 4% of the fecal microbiota (Macchione et al., 2019). Although overall considered a positive commensal, its overgrowth could promote excessive mucin degradation leading to an increase of mucosal permeability (Huang et al., 2019). Reunanen and coworkers (Reunanen et al., 2015) demonstrated that *Akkermansia* can induce a weak pro-inflammatory activity *in vitro*, which usually helps in maintaining host immune response at the mucosal level. The mean relative abundance of *Akkermansia* that we found in the DN subjects was about doubled (8.7%) and returned to normal levels after one year of ASMs. Such an enrichment could participate in local and systemic inflammation that, in turn, could help in seizure triggering inflammation (Rana and Musto, 2018). A recent study on children with refractory epilepsy who did benefit by six months of ketogenic diet, described an initial increase in *Akkermansia* relative abundance that was mitigated by dietary regimen (Gong et al., 2021). According to literature data, gut transit time could impact on *Akkermansia* relative abundance as has been demonstrated that it positively correlates with transit time and stool firmness (Vandeputte et al., 2016; Asnicar et al., 2021). However, we did not observe differences in stool consistency between patients and controls that can sustain the hypothesis of a transit time-related enrichment of this taxon, and other genera associated with a long transit time (i.e., *Bacteroides* spp. and *Alistipes* spp.) were similar in the two groups.

Proteobacteria, and in particular *Escherichia/Shigella* genus, were also found to be increased in the DN subjects compared to healthy controls and gradually reduced by ASM therapy. This observation is in agreement with previous data reporting an increase of this phylum in patients with epilepsy (Xie et al., 2017; Şafak et al., 2020), independently from the drug responsiveness.

The pro-inflammatory activity of *Enterobacteriaceae* is well-described in the literature (Reinoso Webb et al., 2016) and can result from both bacterial structural components (i.e. microbe-associated molecular patterns - MAMPs) such as lipopolysaccharide (LPS) and bacterial metabolism (Ceccarani et al., 2020; Baldelli et al., 2021). Besides *Escherichia/Shigella* genus, the DN subjects had an overall increased abundance in Gram-negative bacteria compared with healthy children. LPS is the main component of their cell wall and, by engaging the TLR4 receptor, it can activate the MyD88-dependent signaling pathway in the lamina propria, resulting in the secretion of proinflammatory mediators that could trigger and perpetuate local inflammation (Ghosh et al., 2020). Moreover, TLR4 signaling has been shown to promote neuroinflammation (Paudel et al., 2020) and LPS administration in rodent models, both locally and systemically, results in seizure induction (Hauss-Wegrzyniak et al., 2000; Maroso et al., 2010).

In the DN subjects, the pro-inflammatory action of Proteobacteria could be exacerbated by the simultaneous decrease of *Lachnospiraceae NK4A136* and *Faecalibacterium* spp., known butyrate-producing bacteria with anti-inflammatory properties (Sokol et al., 2008; Stadlbauer et al., 2020). ASM therapy restored their relative abundance to levels comparable to those of HC. Şafak and colleagues also found a reduction in butyrate-producing taxa in adult patients with focal epilepsy (Şafak et al., 2020) and Lee et al. identified *Faecalibacterium* as indicator species for healthy controls in a case-control study on children with refractory epilepsy (Lee et al., 2021). Of note, the correlation analysis between dietary nutrients and the most abundant bacterial genera revealed for *Lachnospiraceae NK4A136* group strong positive correlations with all of the main macronutrients in the HC group, but none in the DN cohort that showed an almost complete absence of this genus (0.9%). Although not significant, *Faecalibacterium* showed a similar pattern, being positively correlated to dietary intakes in the HC group while negatively in the DN cohort. These preliminary results seem to suggest a complex relationship between diet and gut microbiota that dietary macronutrient intake itself cannot fully explain. The integration with other factors, such as eating behaviors and nutrient provenance, and a more detailed analysis of micronutrients and food additives might shed light on the complex crosstalk between microbial communities and diet (Leeming et al., 2021).

Recent evidence supports a reciprocal causal link between neuroinflammation and epilepsy: proinflammatory cytokines as well as activation of inflammatory pathways in epileptic tissues have been described in both animal models and in humans (Vezzani, 2020). Indeed, patients with drug-resistant seizures may benefit from corticosteroids, ketogenic diet, vagal nerve stimulation, and cannabinoids, which all have mechanisms of action suggesting that the therapeutic effects involve anti-inflammatory mechanisms (Ravizza and Vezzani, 2018). Moreover, the serum levels of specific inflammatory molecules such as HMGB-1, TLR4, IL-1, IL-1R1, and TNF-α, have been recently demonstrated to be higher in children with DRE than in healthy controls, suggesting a possible role as epilepsy biomarkers for these cytokines (Kamaşak et al., 2020).

Our results suggest the possible existence of differences, in terms of general microbial diversity and taxonomy, in the gut microbiota of children at seizure onset. The absence of statistically significant values in the ecological bioestimators alpha- and beta-diversity could be due to the small sample set. In fact, the most relevant limitation of our research is the small number of samples that were analyzed. Due to the unpredictable nature of the seizure onset in the pediatric population, the enrollment of a higher number of drug-naive patients during the recruitment period has been very difficult, and consecutive samples from the same patient are not easy to obtain in real-world settings.

Further studies, carried out on a larger number of patients at different times and disease stage, could contribute to reinforcing these findings in terms of interactions between the microbiota and innate mechanisms of host defense, thus paving the way for the development of new strategies for seizure management (Mejía-Granados et al., 2021).

## Data Availability Statement

Raw reads are available in NCBI Short Read Archive (SRA, http://www.ncbi.nlm.nih.gov/sra) under accession number PRJNA755856.

## Ethics Statement

The study was approved by the Local Ethics Committee (protocol number 2016/ST/199, 28 July 2016). Written informed consent was obtained from the parents or legal guardians of the enrolled patients/healthy subjects. Written informed consent to participate in this study was provided by the participants’ legal guardian/next of kin.

## Author Contributions

EB and AV designed the study. CC, EO, EB, and MS performed experiments and data analysis. IV, MR, and AV performed subject enrollment and analyzed clinical data. CC, EB, and AV performed writing, review, supervision, and editing. All authors discussed the results and commented on the manuscript. All authors contributed to the article and approved the submitted version.

## Funding

This study was supported by the Molecular and Translational Medicine PhD Università degli Studi di Milano scholarship (to EO and CC).

## Conflict of Interest

The authors declare that the research was conducted in the absence of any commercial or financial relationships that could be construed as a potential conflict of interest.

## Publisher’s Note

All claims expressed in this article are solely those of the authors and do not necessarily represent those of their affiliated organizations, or those of the publisher, the editors and the reviewers. Any product that may be evaluated in this article, or claim that may be made by its manufacturer, is not guaranteed or endorsed by the publisher.
