# Supplementary figures and images for: Is Gut Microbiota a Key Player in Epilepsy Onset? A Longitudinal Study in Drug-Naive Children
Source: Front Cell Infect Microbiol. 2021 Dec 3;11:749509. doi: 10.3389/fcimb.2021.749509 (PMC8677705; doi:10.3389/fcimb.2021.749509)

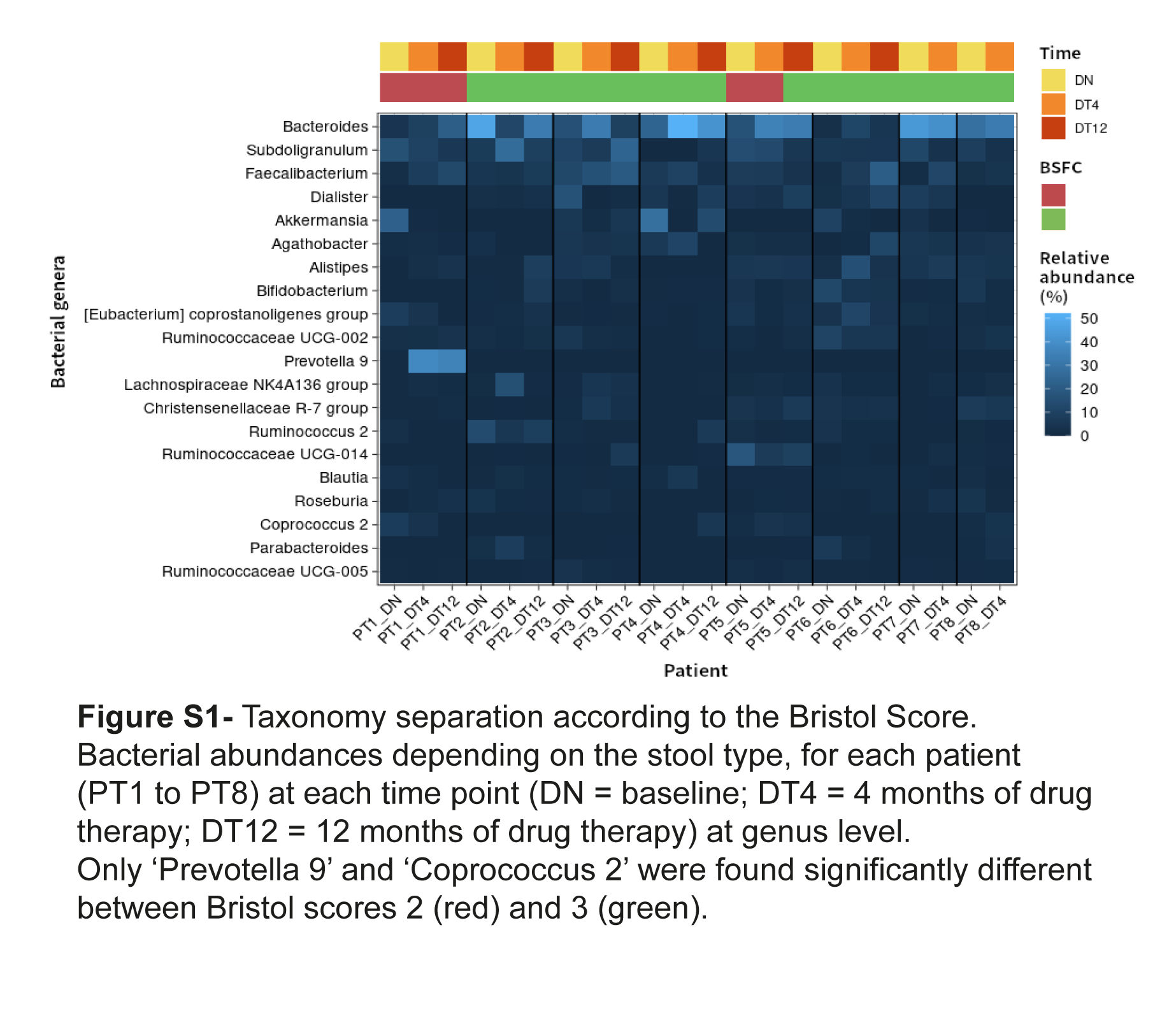

Supplement: Supplementary file 1 [file Image_1.tif]

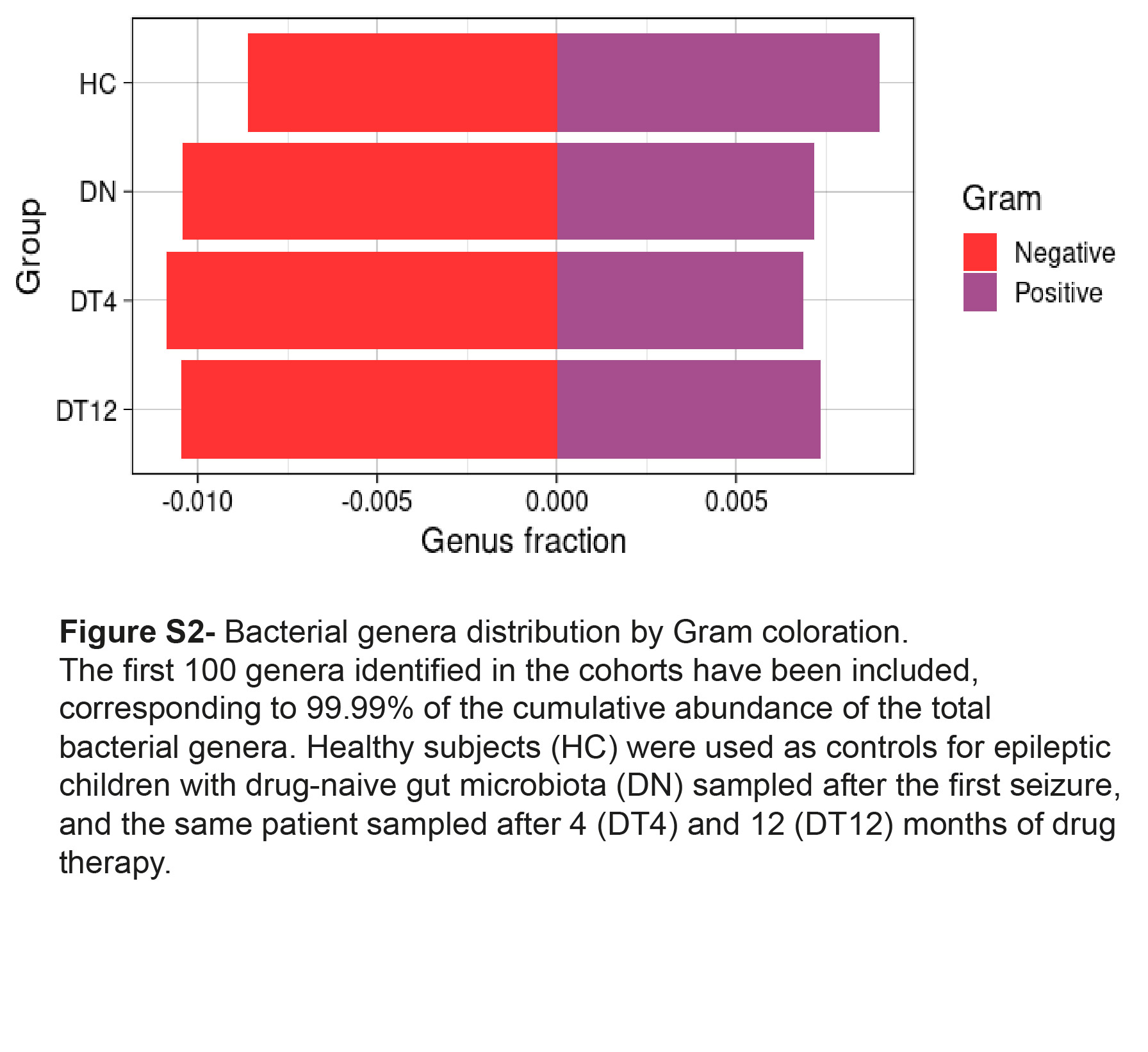

Supplement: Supplementary file 2 [file Image_2.jpeg]

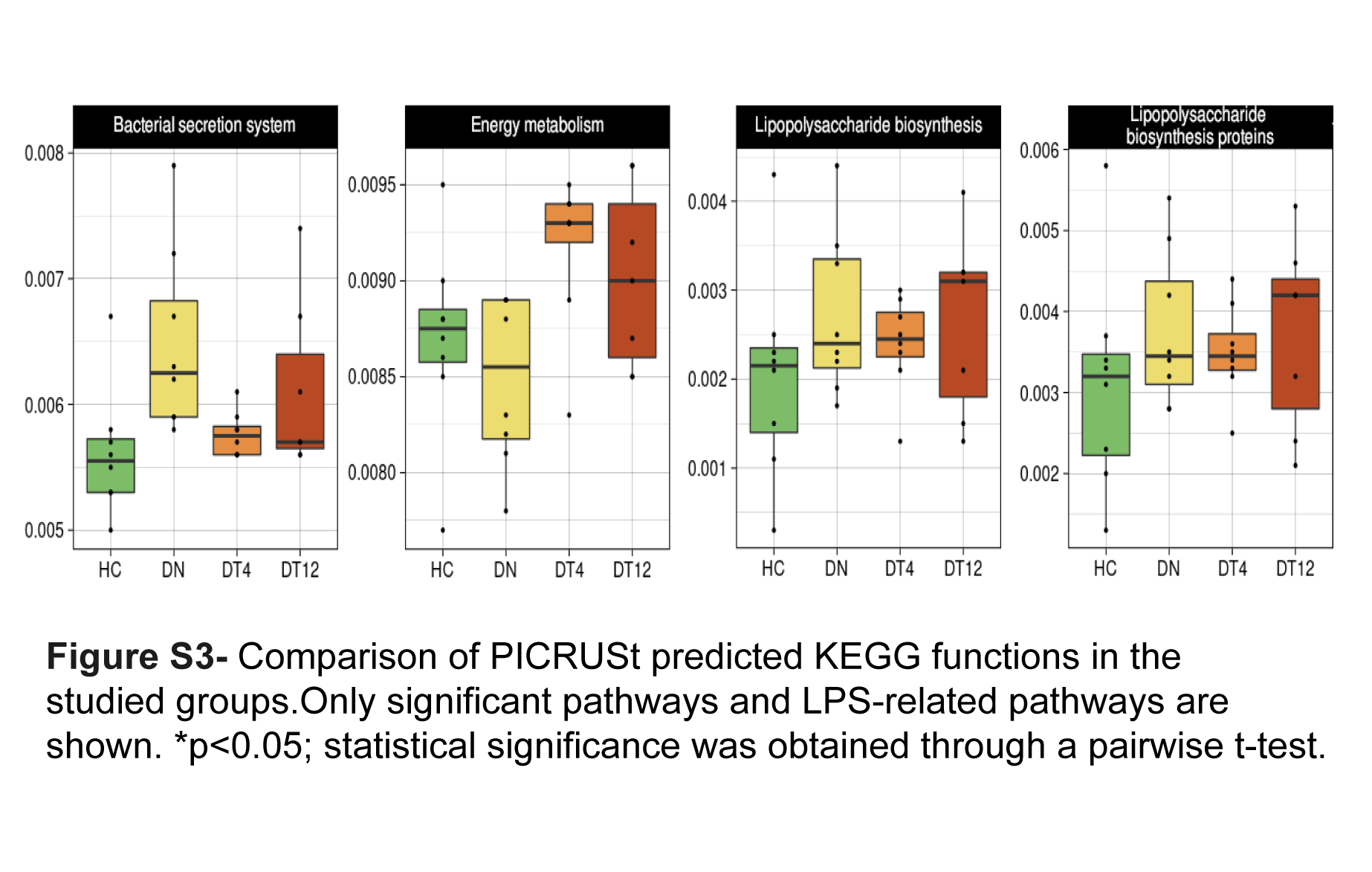

Supplement: Supplementary file 3 [file Image_3.tif]
