# Supplementary material for: Is Gut Microbiota a Key Player in Epilepsy Onset? A Longitudinal Study in Drug-Naive Children
Source: Front Cell Infect Microbiol. 2021 Dec 3;11:749509. doi: 10.3389/fcimb.2021.749509 (PMC8677705; doi:10.3389/fcimb.2021.749509)
Supplement: Supplementary file 4 [file Table_1.docx]

**Table S1. Clinical characteristics of children with new-onset epilepsy included in the study**

|  | **Gender** | **Delivery mode** | **Age (y) at epilepsy onset** | **Seizure frequency at onset** | **Epilepsy diagnosis** | **Therapy** | **ASM plasmatic level (mg/mL)** | | **Seizure frequency at follow up** | | **Bristol**  **Stool Form Scale** |
| --- | --- | --- | --- | --- | --- | --- | --- | --- | --- | --- | --- |
|  |  |  |  |  |  |  | **DT4** | **DT12** | **DT4** | **DT12** |  |
| P1 | F | Vaginal | 8 | Daily | Childhood Absence Epilepsy | Valproic Acid | 79 | 83 | 0 | 0 | 2 |
| P2 | M | Vaginal | 13 | Monthly | Focal Epilepsy | Valproic Acid | 73 | 76 | 0 | 0 | 3 |
| P3 | F | Assisted Vaginal* | 15 | Weekly | Adolescence Absence Epilepsy | Valproic Acid | 50 | 51 | 0 | 0 | 3 |
| P4 | F | Vaginal | 3 | Monthly | Focal Epilepsy | Levetiracetam | 5.9 | 6.2 | 0 | 0 | 3 |
| P5 | F | Caesarean | 9 | Daily | Absence Epilepsy | Ethosuximide | 44.9 | 45.5 | sporadic | 0 | DN: 2  DT12: 3 |
| P6 | F | Vaginal | 9 | Daily | Absence Epilepsy | Valproic Acid | 85.5 | 91 | 0 | 0 | 3 |
| P7 | F | Cesarean | 3 | Monthly | Focal Epilepsy | Valproic Acid | 64 | 68 | 0 | 0 | 3 |
| P8 | M | Vaginal | 9 | 6-7/year | Focal Epilepsy | Carbamazepine | 9.4 | 8 | 0 | 0 | 3 |

*vacuum device; DT4 = 4 months follow up; DT12 = 12 months follow up
