# Supplementary material for: Is Gut Microbiota a Key Player in Epilepsy Onset? A Longitudinal Study in Drug-Naive Children
Source: Front Cell Infect Microbiol. 2021 Dec 3;11:749509. doi: 10.3389/fcimb.2021.749509 (PMC8677705; doi:10.3389/fcimb.2021.749509)
Supplement: Supplementary file 5 [file Table_2.docx]

**Table S2. Daily dietary intake of energy and macronutrients in patients and healthy controls.**

| **Variable** | **HC**  **Mean (SD)** | **DN**  **Mean (SD)** | **p-value** | **Reference values*** |
| --- | --- | --- | --- | --- |
| **Energy intake**  Kcal  from soft drinks  from yogurt | 1018 (463)  50.4**^#^**  128**^#^** | 1351 (516)  101,8 (36.22)  43**^#^** | ns | boys:1330-4020  girls:1220-3550  kcals (AR) |
| **Proteins**  g  % Energy  From vegetables, g  From animal source, g | 42.71 (19.03)  17.1 (1.82)  9.13 (3.48)  24.2 (4.5) | 55.56 (17.36)  17.96 (3.13)  13.71 (5.24)  23.74 (9.73) | ns | 16-50 g (AR)  12-15% (RI)  boys: 0.76e0.79 g/kg (AR)  girls: 0.76e0.77 g/kg (AR) |
| **Lipids**  g  % energy | 37.88 (19.29)  33.25 (4.6) | 46.31 (8.2)  32.05 (8.75) | ns | 20-35% (RI) |
| **Carbohydrates**  g  % Energy  Simple sugars, g  Starch, g | 131.14 (55.63)  49.68 (3.25)  52.19 (20.74)  51.65 (14.50) | 167.73 (53.69)  50.52 (8.71)  40.95 (24.98)  68.98 (33.95) | ns | 45-60% (RI) |
| **Total fiber**  g  g/1000 Kcal  Insoluble fiber, g  Soluble fiber, g | 8.04 (5.22)  7.66 (2.00)  3.1 (1.96)  1.12 (0.46) | 9.80 (3.04)  9.28 (3.34)  3.73 (2.81)  1.11 (0.51) | ns | 8.40 g/1000 kcal (AI) |

HC = healthy controls; DN = children with epilepsy before drug therapy onset; AR = average requirement; RI = reference intake; AI = adequate intake.

**^#^**one child per group (no SD). *Reference values: Società Italiana di Nutrizione Umana (Italian Society of Human Nutrition) , SINU. Nutrients and energy reference intake levels for italian population; IV revision. Milan, Italy: Società Italiana di Nutrizione Umana; 2014). Values are expressed as mean (standard deviation). P-values <0.05 are considered significant (Mann-Whitney test); ns = non-statistically significant.
