# Supplementary material for: Is Gut Microbiota a Key Player in Epilepsy Onset? A Longitudinal Study in Drug-Naive Children
Source: Front Cell Infect Microbiol. 2021 Dec 3;11:749509. doi: 10.3389/fcimb.2021.749509 (PMC8677705; doi:10.3389/fcimb.2021.749509)
Supplement: Supplementary file 6 [file Table_3.docx]

**Table S3. Bacterial composition.** Relative abundances among cohorts are reported as mean (standard deviation).

| **Phylogenetic level** | | | **HC** | **DN** | **DT4** | **DT12** | **p-value** (raw) |  |
| --- | --- | --- | --- | --- | --- | --- | --- | --- |
| **Phylum** | **Family** | **Genus** |  |  |  |  |  | |
| Firmicutes |  |  | 66.4 (17.7) | 56.8 (13.2) | 53.4 (11.9) | 57.7 (18.7) |  | |
|  | *Ruminococcaceae* |  | 38.7 (12.7) | 30.9 (13.2) | 29.9 (11.7) | 36.6 (14.4) |  | |
|  |  | *Faecalibacterium* | 11 (11.6) | 4.7 (3.8) | 8.6 (4.0) | 11.1 (7.8) |  | |
|  |  | *Ruminococcaceae UCG-002* | 3 (2.4) | 3 (3.4) | 1.9 (1.8) | 2.5 (1.6) |  | |
|  |  | *Ruminococcus 2* | 1.7 (1.2) | 3 (4.4) | 1 (1.7) | 3.1 (3.7) |  | |
|  |  | *Ruminococcaceae UCG-014* | 1.4 (1.9) | 2.5 (6.4) | 1.4 (2.4) | 2.8 (4.1) |  | |
|  |  | *Ruminococcus 1* | 1.3 (1.5) | 0.9 (1.2) | 0.8 (0.9) | 1.1 (1.3) |  | |
|  | *Lachnospiraceae* |  | 19.1 (8.0) | 14.9 (6.3) | 14.9 (6.3) | 11.2 (4) | HC-DT12 0.025 | |
|  |  | *Lachnospiraceae NK4A136 group* | 4.1 (4.7) | 0.9 (1.0) | 3.5 (4.9) | 1.2 (0.9) |  | |
|  |  | *Agathobacter* | 3.6 (2.8) | 3.2 (2.0) | 3.4 (3.3) | 3.2 (4.6) |  | |
|  |  | *Roseburia* | 1.4 (1.4) | 1.3 (1.7) | 1.1 (0.8) | 1 (0.9) |  | |
|  |  | *Blautia* | 2.5 (4.8) | 1.2 (0.7) | 1.5 (1.8) | 0.7 (0.4) |  | |
|  | *Veillonellaceae* |  | 3.5 (3.2) | 6 (5.8) | 6 (5.8) | 5.7 (4.8) |  | |
|  | *Christensenellaceae* |  | 2.7 (2.8) | 2.2 (2.7) | 2.2 (2.7) | 2.4 (2.4) |  | |
|  |  | *Christensenellaceae R-7 group* | 2.6 (2.8) | 2.1 (2.6) | 2.3 (2.5) | 2.3 (2.4) |  | |
|  | *Acidaminococcaceae* |  | 0.4 (0.6) | 1.1 (1.3) | 1.1 (1.3) | 0.2 (0.3) |  | |
|  | Other families | *Subdoligranulum* | 10.4 (7.0) | 9.3 (5.1) | 8.5 (8.3) | 8.9 (7.5) |  | |
|  |  | *Dialister* | 3.1 (3.2) | 4.8 (5.4) | 1.9 (2.1) | 5.3 (4.5) |  | |
|  |  | *[Eubacterium] coprostanoligenes group* | 2.7 (1.9) | 2.6 (2.6) | 2.6 (3.7) | 2 (1.6) | HC-DN 0.004;  HC-DT12 0.008 | |
| Bacteroidetes |  |  | 27.5 (15.6) | 29 (17.5) | 42 (11.4) | 34.8 (19.6) |  | |
|  | *Bacteroidaceae* |  | 21.5 (14.5) | 22.8 (16.7) | 22.8 (16.7) | 23.7 (14.5) |  | |
|  |  | *Bacteroides* | 21.5 (14.5) | 22.8 (16.7) | 28.1 (15.7) | 23.7 (14.5) |  | |
|  | *Rikenellaceae* |  | 2.9 (2.3) | 2.9 (2.3) | 2.9 (2.3) | 3.7 (3.0) |  | |
|  |  | *Alistipes* | 2.9 (2.2) | 2.9 (2.3) | 5.2 (4.8) | 3.7 (3.0) |  | |
|  | *Tannerellaceae* |  | 0.8 (0.7) | 1.3 (2.2) | 1.3 (2.2) | 0.3 (0.5) |  | |
|  | *Prevotellaceae* |  | 0.7 (1.4) | 0.5 (0.9) | 0.5 (0.9) | 6 (14.4) |  | |
|  |  | *Prevotella 9* | 0.0 (0.0) | 0.1 (0.3) | 4.7 (13.3) | 5.9 (14.4) |  | |
| Actinobacteria |  |  | 3.1 (5.4) | 3.4 (4.2) | 1.2 (1.5) | 2.9 (2.6) |  | |
|  | *Bifidobacteriaceae* |  | 2.9 (5.3) | 3.2 (4.0) | 3.2 (4.0) | 2.8 (2.5) |  | |
|  |  | *Bifidobacterium* | 2.9 (5.3) | 3.2 (4.0) | 1.1 (1.4) | 2.8 (2.5) |  | |
| Verrucomicrobia |  |  | 2.1 (3.5) | 8.7 (10.8) | 0.5 (0.5) | 3.2 (5.5) | HC-DN 0.028;  DN-DT4 0.012 | |
|  | *Akkermansiaceae* |  | 2.1 (3.5) | 8.7 (10.8) | 0.5 (0.5) | 3.2 (5.5) | HC-DN 0.028;  DN-DT4 0.012 | |
|  |  | *Akkermansia* | 2.1 (3.5) | 8.7 (10.8) | 0.5 (0.5) | 3.2 (5.5) | HC-DN 0.028;  DN-DT4 0.012 | |
| Proteobacteria |  |  | 0.5 (0.5) | 1.9 (2.0) | 2.9 (2.2) | 1.3 (0.7) | HC-DT4 0.002 | |
|  |  | *Escherichia-Shigella* | 0.1 (0.3) | 1.0 (1.3) | 0.8 (1.2) | 0.5 (0.6) | HC-DN 0.046 | |

Statistical differences were computed through a non-parametic Mann-Whitney U-test; p-values <0.05 were considered significant.
